# Seasonal and geographic variation in packed cell volume and selected serum chemistry of platypuses

**DOI:** 10.1038/s41598-021-95544-z

**Published:** 2021-08-05

**Authors:** Jana Stewart, Gilad Bino, Tahneal Hawke, Richard T. Kingsford

**Affiliations:** Centre for Ecosystem Science, School of Biological, Earth & Environmental Sciences, UNSW, Sydney, NSW 2052 Australia

**Keywords:** Biochemistry, Ecology, Zoology, Ecology, Environmental sciences, Biomarkers, Signs and symptoms

## Abstract

Platypuses (*Ornithorhynchus anatinus*) inhabit the permanent rivers and creeks of eastern Australia, from north Queensland to Tasmania, but are experiencing multiple and synergistic anthropogenic threats. Baseline information of health is vital for effective monitoring of populations but is currently sparse for mainland platypuses. Focusing on seven hematology and serum chemistry metrics as indicators of health and nutrition (packed cell volume (PCV), total protein (TP), albumin, globulin, urea, creatinine, and triglycerides), we investigated their variation across the species’ range and across seasons. We analyzed 249 unique samples collected from platypuses in three river catchments in New South Wales and Victoria. Health metrics significantly varied across the populations’ range, with platypuses from the most northerly catchment, having lower PCV, and concentrations of albumin and triglycerides and higher levels of globulin, potentially reflecting geographic variation or thermal stress. The Snowy River showed significant seasonal patterns which varied between the sexes and coincided with differential reproductive stressors. Male creatinine and triglyceride levels were significantly lower than females, suggesting that reproduction is energetically more taxing on males. Age specific differences were also found, with juvenile PCV and TP levels significantly lower than adults. Additionally, the commonly used body condition index (tail volume index) was only negatively correlated with urea, and triglyceride levels. A meta-analysis of available literature revealed a significant latitudinal relationship with PCV, TP, albumin, and triglycerides but this was confounded by variation in sampling times and restraint methods. We expand understanding of mainland platypuses, providing reference intervals for PCV and six blood chemistry, while highlighting the importance of considering seasonal variation, to guide future assessments of individual and population condition.

## Introduction

Body condition, morphology^1^, body mass^2,3^, skeletal size^4,5^, pelage^6,7^, and behaviour of many animal species vary across their ranges^8,9^. Mechanisms causing these variations are complex and differ among species^10–12^ but often reflect responses to climatic drivers, such as temperature and seasonality, altering niche quality^13–17^. Threatening processes, particularly those that alter the environment, can also impact a species’ realized niche^18^, affecting morphology^19^, breeding cycles^20^, and sometimes body condition of animals, resulting from poor nutrition or increased parasite load^21–23^.

The viability of a wild population is dependent on a range of life history characteristics such as life expectancy and reproductive rate. These life history characteristics can be adversely affected by poor individual health within the population. Level of nutrition is an important determinant of individual health^24–26^. Information on health or nutrition levels often relies on measuring surrogates of body condition such as mass or other morphological indices on wild animals, but these may not adequately measure health of individuals^27,28^. Morphological indices are useful field measurements, but many are subjective and often lack standardisation for assessment. Body condition is directly linked to nutritional status, with animals storing surplus energy in tissues and organs (e.g., fat deposits, kidney and liver) at times of high food availability and/or low metabolic need, mobilizing these stores at times of low food availability and/or high metabolic need. Hematology and serum chemistry analytes are useful to measure body condition and organ function^29,30^, and can detect more subtle variations in health that morphological indices might not detect. They are particularly valuable for endangered and difficult to study species. They can be used to investigate effects of environmental stressors^31,32^, underlying disease and parasite burden^33,34^, and reproductive cycles^35,36^. For example, packed cell volume (PCV), red blood count, white blood count, hemoglobin, urea, creatinine, triglycerides and albumin were used to identify a malnourished sub-population of Tammar wallaby (*Macropus eugenii*), in poorer condition than neighboring sub-populations^37^. Further, hematocrit, hemoglobin, red blood cell count, creatine, and triglycerides were used to effectively measure condition and effects of stressors over time in relocated eastern bettongs^38^. These cases demonstrate how hematology and serum chemistry can provide valuable population information not otherwise detected with physical examination alone.

PCV is the percentage of the blood volume that is made up of red blood cells, which carry oxygen and drive metabolism^39^. PCV has a complex relationship to body condition and stress responses. Commonly in wildlife, elevations may indicate dehydration or acute stress while decreases are often associated with anaemia. Alone its interpretation can be difficult, but it can be used with other variables to estimate response to stressors such as food availability^40^, seasons^41,42^, and breeding^43^. Serum chemistry analytes, including total protein, albumin, globulin, urea, creatinine, and triglycerides, measure proteins and fats, and are associated with energy and protein balance^29,44,45^. Proteins are critical for muscle mass and organ function. Relatively lower levels can reflect poor nutrition, and reduced muscle mass^29,44^. Further, low levels of albumin can reflect an acute phase protein response^46^, while high levels of globulin can reflect inflammation^47^. Urea and creatinine primarily indicate kidney dysfunction when elevated^48,49^. Protein intake can be measured with total protein, albumin, and urea, useful for measuring short term nutrition, while creatinine levels indicate long-term nutrition^44,50–53^. Triglycerides correlate to fat stores, generally used first before protein stores, indicating energy available for metabolic demands, with low levels reflecting recent metabolic stress^47^. For free-ranging wildlife their value for providing richer information on population health and its potential correlations with environmental stress which is otherwise difficult measure. Importantly, establishing species and population specific Reference Intervals (RI) for health data provides a quantifiable method to assess population specific stressors and understanding of seasonal variation^54,55^. This was highlighted in a recent study of eastern grey kangaroos (*Macropus giganteus*)^56^ which found a significant number of animals from a high density population were outside of the RI’s for the species, indicating the health impact of the increased population density which can then be used to inform management of the population. The international standard for reliable representation of a population requires 120 individuals^57,58^, which can be difficult to achieve. Consequently, few species have established baseline data.

There are a range of factors that might cause variation in hematology and serum chemistry analytes between platypus populations. Platypuses have four genetically distinct populations across their distribution in Australia: Tasmania, Victoria and New South Wales combined, Queensland, and King Island^59^. They also vary in morphology across their range including: body mass (610–1500 g in Queensland to 1200–3000 g in Tasmania^60^) and body length (Tasmanian individuals 21–29% longer than on the mainland^61^). These differences reflect Bergmann’s and Allen’s rules ^62,63^ of adaptation to decreasing temperature with latitude (20°–32 °C Queensland, 9°–25 °C Tasmania). Climatic influence on breeding seasons can be seen across their range also, with courtship followed by nesting behaviour beginning in August in New South Wales while in Tasmania it is starts in October^64–66^. Habitats also vary across the platypuses’ range, with differences in their food of aquatic macroinvertebrates^67–71^. Platypus habitat quality and health are under increasing pressure from anthropogenically driven stressors, including water resource development lowering water availability and altering the natural flow regime, land clearing destroying riparian vegetation, bank erosion and sedimentation, as well as pollution, all of which vary in largely unquantified severity across their range^72–78^. The health of the Tasmanian population is increasingly understood in relation to sex, disease, and seasonal variation^79–81^. However all three studies on the New South Wales and Victorian population have had insufficient sample sizes^82–84^, n =  < 50 (except Booth and Connolly^82^with n = 87 for PCV), to meet the accepted international standard of 120 for reliable representation of a population.

We aimed to add to the sparse health data and establish reference intervals for mainland platypuses and for the species in general. Using pathology parameters that have been associated with body condition and nutrition in other wildlife species, we evaluated parameters of platypus health related to energy and protein. We investigated hematology and serum chemistry analytes from three studied areas in New South Wales and Victoria, examining variation among seasons, sexes, age, and elevation. Specifically, we tested for differences in PCV, TP, albumin, globulin, urea, creatinine, and triglycerides levels. We predicted that hematology and serum chemistry analytes from mainland platypuses would follow a similar pattern to that observed for PCV and albumin in Tasmanian individuals previously studied, increasing in winter and decreasing in summer^85^, and varying between river catchments^80^, sex, and age^85^. We also examined the association between hematology and serum chemistry analytes and Tail Volume Index (TVI)^85^, the most commonly used field body condition metric for platypuses. Finally, we reviewed available literature and evaluated for any biogeographic variation in examined serum chemistry analytes and PCV across the species’ range.

## Methods

### Field samples

Between January 2016 and May 2018, 259 platypuses were trapped using fyke and gill nets, and anaesthetized using isoflurane gas prior to inspection and collection of blood^86,87^. Platypuses were surveyed in three river catchments across seven rivers in New South Wales; Border Rivers Catchment (Tenterfield Creek n = 42, and Severn River n = 41, Jan–May 2016), Snowy Rivers Catchment (Eucumbene n = 26, Snowy n = 90, Thredbo Rivers n = 22, Dec 2016-Nov 2017); and Victoria, Upper Murray Rivers Catchment (Mitta Mitta n = 18, and Ovens Rivers n = 20, Jan–May 2018, Fig. 1). Elevation was calculated using a one second Digital Elevation Models^88^ for each sample (Severn 393 m-819 m, Tenterfield 373 m-816 m, Eucumbene 939 m-1336 m Snowy 745 m-847 m, Thredbo 921 m-1352 m, Mitta Mitta 282-m541m, Ovens 244 m-405 m). Body condition of each platypus was evaluated, inspecting for external injuries and fat content using tail volume index (TVI, 1–5 with 1 indicating high levels of fat stored in the tail and 5 indicating low levels), a commonly used qualitative measure of fat reserves^85^. No individuals presented with apparent diseases or injuries. Sex and age (Juvenile or Adult) were also determined, based on presence and shape of spurs^89,90^. Blood samples of 2 mL were collected from the bill sinus^83^. Packed cell volume (PCV, %) and total protein (TP, g/L) were determined using a microhematocrit heparinized capillary tube and refractometer, following centrifugation (75 mL, 10,000 g, 3 min), with the remaining blood centrifuged (2000 g) for 10 min to separate serum, which was then stored at -80° C. Chilled serum was analyzed in 2019 by Vetnostics Laboratory, using a Cobas 8000 (Roche diagnostic systems), providing blood serum chemistry data for albumin (g/L), urea (mmol/L), creatinine (umol/L), and triglycerides (mmol/L). Globulin (g/L) was determined by subtracting albumin from the TP.

**Figure 1:**
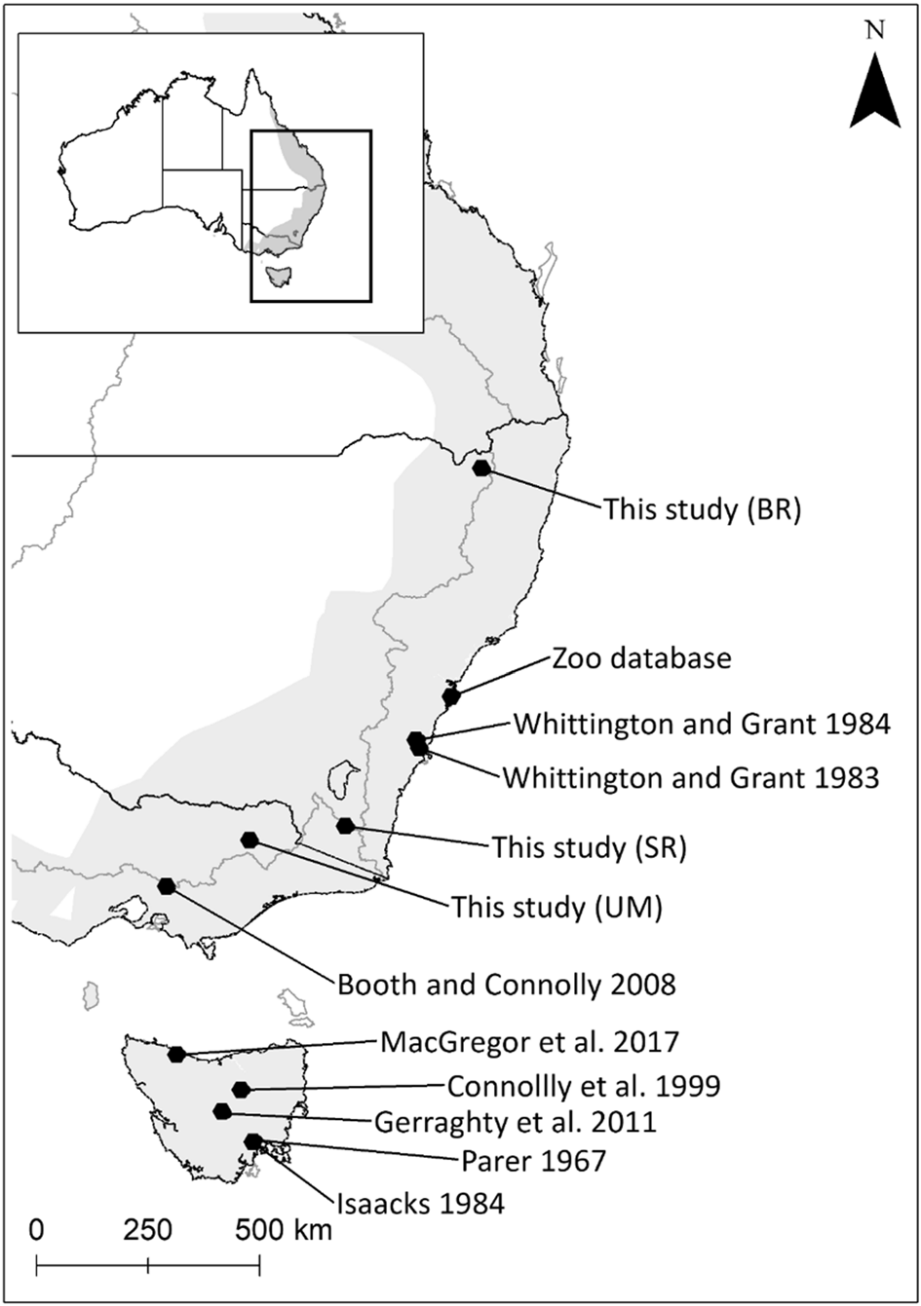
IUCN distribution of platypuses (grey shading in insert and main figure) and sampling locations for this study (*BR* Border Rivers, *SR* Snowy River, *UM* Upper Murray River) and other published or unpublished studies (see Table 1 for numbers related to publications and other available sources).

### Analytical approach

We predicted that hematology and serum chemistry analytes from platypus in New South Wales and Victoria would follow a similar pattern to that observed for PCV and albumin in Tasmanian platypuses, increasing in winter and decreasing in summer^81^. We examined associations between seven dependent hematology and serum chemistry response variables (PCV, TP, albumin, globulin, urea, creatinine, and triglycerides) and six independent explanatory variables: month (Jan-May, continuous 1 to 5), catchment (three levels), elevation of sample, sex (female/male), age (adult/juvenile), and tail volume index. We evaluated a linear response to month, assuming a general trend between Jan and May^81^ while accounting for non-linear responses in addition to an interaction term with sex. We fitted a Generalized Linear Model (GLM) assuming a Gaussian error distribution, examining all possible predictor combinations of the seven response variables (n = 64), and ranked model performance, based on the corrected Akaike Information Criterion (AICc)^91^. All dependent variables were initially checked for normality using the Shapiro–Wilk Normality Test before analysis. Appropriateness of linear models were also visually evaluated using the residuals vs fitted plots to check homogeneity and linear relation, along with normal q-q plots to check normal distribution of residuals. No data required transformation. To address model uncertainty, we considered the full model space, examining all possible explanatory variable combinations to identify the best fit models. As a measure of model fit, we used the corrected Akaike’s Information Criterion (AICc) and considered those having AICc scores not greater than four relative to the lowest score^92^. We then applied a model averaging approach, weighting predictor effect sizes by model AICc scores, to quantify the average strength of association between response variables and examined predictors. Associations were considered significant if *P* ≤ 0.05. We used the ‘dredge’ and ‘model.avg’ functions in the ‘MuMIn’ package within the R environment^93^. The ‘ggplot2′^94^ package was used to represent the data.

Additional surveys undertaken on the Snowy River between Jindabyne Dam and the town of Dalgety (~ 20 km)^87^, carried out of between December 2016 to November 2017, allowed us to investigate seasonal variation in adult hematology and serum chemistry parameters, along with TVI, between sexes over a full annual cycle. We used a Generalized Additive Model (GAM), assuming a Gaussian error distribution fitted, with a cyclical relationship (cyclic cubic spline) with month as a continuous variable (in concordance with sampling sequence) and an interaction term for sex (n = 82). To allow for seasonal variation with a single maxima and minima and avoid over-fitting, we limited the number of knots (i.e. polynomial level) to three. Associations were considered significant if *P*  ≤ 0.05. We used the ‘gam’ function in the ‘mgcv’ package^95,96^ in the R^93^. The ‘visreg’^97^ package was used to represent the data.

We used samples collected in this study to establish reference intervals for New South Wales and Victorian platypuses in our three study catchments using Reference Value Advisor v2.1^98^, and the standards set by the American Society for Veterinary Clinical Practice^99^. Using non-parametric methods, as recommended for sample sizes over 120^98^, we calculated the 95th percentile confidence range and used the 2.5 and 97.5 percentile as the lower and upper limits, and the 90th percentile confidence range as the upper and lower ranges within the reference interval. For reference intervals calculated for individual catchments, sex, and age where sample sizes were between 40 and 120, we estimated reference interval using a non-parametric bootstrapping method^98,99^. In the few cases where sample size was less than 40 a robust method with a Box-Cox transformation of the data was used to provide reference interval^98^.

### Review of published hematology and serum chemistry

We performed a literature search for publications relating to platypus hematology and serum chemistry, using search engines and databases^100^, including: in Google Scholar, Ovid, Scopus and Web of Science. We searched for publications with the terms: ‘platypus hematology’, ‘platypus serum chemistry’, ‘platypus biochemistry’. We identified 11 sources, including eight peer reviewed papers and four book chapters but removed duplicated results, leaving a total of eight sources with hematology and serum chemistry data from Tasmania, Victoria, and New South Wales. In addition, we sourced unpublished data from the ZIMS species360 database (www.species360.org), a global online database used by zoos for captive animals. In total, we used 12 different platypus studies and locations, including the three catchments that we sampled in this study (Fig. 1 and Table 1). To investigate whether hematological or blood chemistry analytes varied non-linearly across the species’ latitudinal range, we used the mean for each analyte and a Generalized Linear Model weighted by sample size^101^, fitted with a second order polynomial relationship, to allow a non-linear association, with latitude as a predictor variable, implemented in R^93^. Associations were considered significant if *P*  ≤ 0.05. The ‘ggplot2′^94^ package was used to represent the data. To further future research into population health of platypus, our data is made open source via Dryad (https://doi.org/10.5061/dryad.brv15dv9d).

**Table 1:** Identified studies with reported platypus hematology and serum chemistry values (location number refers to location on Fig. 1).

#Location	Source	Season\Month	PCV	TP	Albumin	Globulin	Urea	Creatinine	Triglycerides	Lat
1. Inglis River (TAS)	Macgregor, et al.^81^	Annual	0.46 ± 0.04 (0.36–0.57) n = 126	65 ± 4.2 (55.3–77) n = 109	31.1 ± 2.2 (24.9–34.9) n = 109	34 ± 3 (27–44) n = 108	28.7 ± 3.1 (18.1–37.6) n = 108	34 ± 8 (15–59) n = 113	N/A	− 41.05
2. Tasmania	Geraghty, et al.^80^	Jan-Jun	N/A	80.2 ± 28.4 n = 116	21.5 ± 30.4 n = 31	65.3 ± 42.7 n = 29	33.9 ± 31.3 n = 32	30.1 ± 40 n = 33	N/A	− 43.05
3. Brumbys Creek (TAS)	Connolly, et al.^79^	N/A	0.48 ± 0.04 (0.40–0.59) n = 27	N/A	27.2 ± 2.6 (22.5–33.8) N = 27	47 ± 5.4 (34.3–56) n = 27	31.6 ± 2.9 (27–36.4) n = 27	35.2 ± 5 (25–44) n = 27	N/A	− 41.75
4. Kangaroo River (NSW)	Whittington and Grant^84^	Nov	0.49 ± 0.05 n = 9	72 ± 2 n = 4	32 ± 1 n = 4	N/A	31.5 ± 1.7 n = 6	26 ± 1.4 n = 2	2.89 ± 0.55 (1.79–3.99) n = 4	− 34.71
5. Upper Shoalhaven River (NSW)	Whittington and Grant^83^	Jan/Mar	0.52 ± 0.01 n = 29	68 ± 0.8 n = 24	32 ± 0.4 n = 24	N/A	28.9 ± 0.08 n = 24	35 ± 2.2 n = 20	1.58 ± 0.13 (1.32–1.84) n = 24	− 34.86
6. Healesville (VIC)	Booth and Connolly^82^	N/A	0.51 (0.35–0.62) n = 87	66 (57–75) n = 40	28 (22–33) n = 39	37.5 ± 5.25 (25–46) n = 40	N/A	30 (10–40) n = 38	0.87 (0.50–1.5) n = 28	− 37.65
7. Tasmania	Parer and Metcalfe^165^	N/A	0.52 (0.50–0.54) n = 3	N/A	N/A	N/A	N/A	N/A	N/A	− 42.87
8. Tasmania	Isaacks, et al.^166^	N/A	0.43 ± 0.07 (0.29–0.57) n = 10	N/A	N/A	N/A	N/A	N/A	N/A	− 42.47
9. Taronga Zoo (NSW)	Zims 2020	N/A	0.50 (0.38–0.60) n = 22	72 (57–90) n = 22	N/A	N/A	26.8 (16.3–39.6) n = 22	79 (4–124) n = 20	N/A	− 33.84
10. Border Rivers (NSW)	This paper	Dec–May	0.44 ± 0.04 (0.31–0.54) n = 63	68.3 ± 0.6 (44–84) n = 63	31 ± 8 (11–42) n = 57	37.4 ± 14 (11–67) n = 57	30.6 ± 4.2 (15.9–38.7) n = 57	49.1 ± 16.8 (15–90) n = 47	0.67 ± 0.41 (0.2–2.1) n = 43	− 29.24
11. Snowy River (NSW)	This paper	Annual	0.50 ± 0.05 (0.30–0.60) n = 106	65.74 ± 4.2 (57–82) n = 104	34 ± 4 (24–44) n = 106	31.1 ± 6.3 (23–48) n = 97	29.7 ± 3.7 (20.4–36.7) n = 106	35.10 ± 14.4 (15–75) n = 97	1.25 ± 0.55 (0.50–3.10) n = 105	− 36.44
12. Upper Murray River (VIC)	This paper	Jan–May	0.53 ± 0.04 (0.43–0.60) n = 22	66.7 ± 5.4 (58–78) n = 21	36 ± 12 (30–41) n = 21	33.6 ± 13.3 (17–70) n = 21	30.7 ± 3.1 (26.6–37.2) n = 21	35.9 ± 12.0 (20–70) n = 17	1.49 ± 1.23 (0.7–5.0) n = 21	− 36.72

Values are listed as mean ± SD (min to max), with sample size for particular analyte listed. N/A – unavailable data.

### Guidelines and regulations

Guidelines and Regulations This study was performed in accordance with the guidelines set out and approved by the by the NSW Office of Environmental Heritage (SL101655), NSW Department of Primary Industries (P15/0096=1.0 & OUT15/26392), and UNSW's Animal Care and Ethics Committee (16/14A). This study complies with ARRIVE guidelines.

## Results

**Figure 2:**
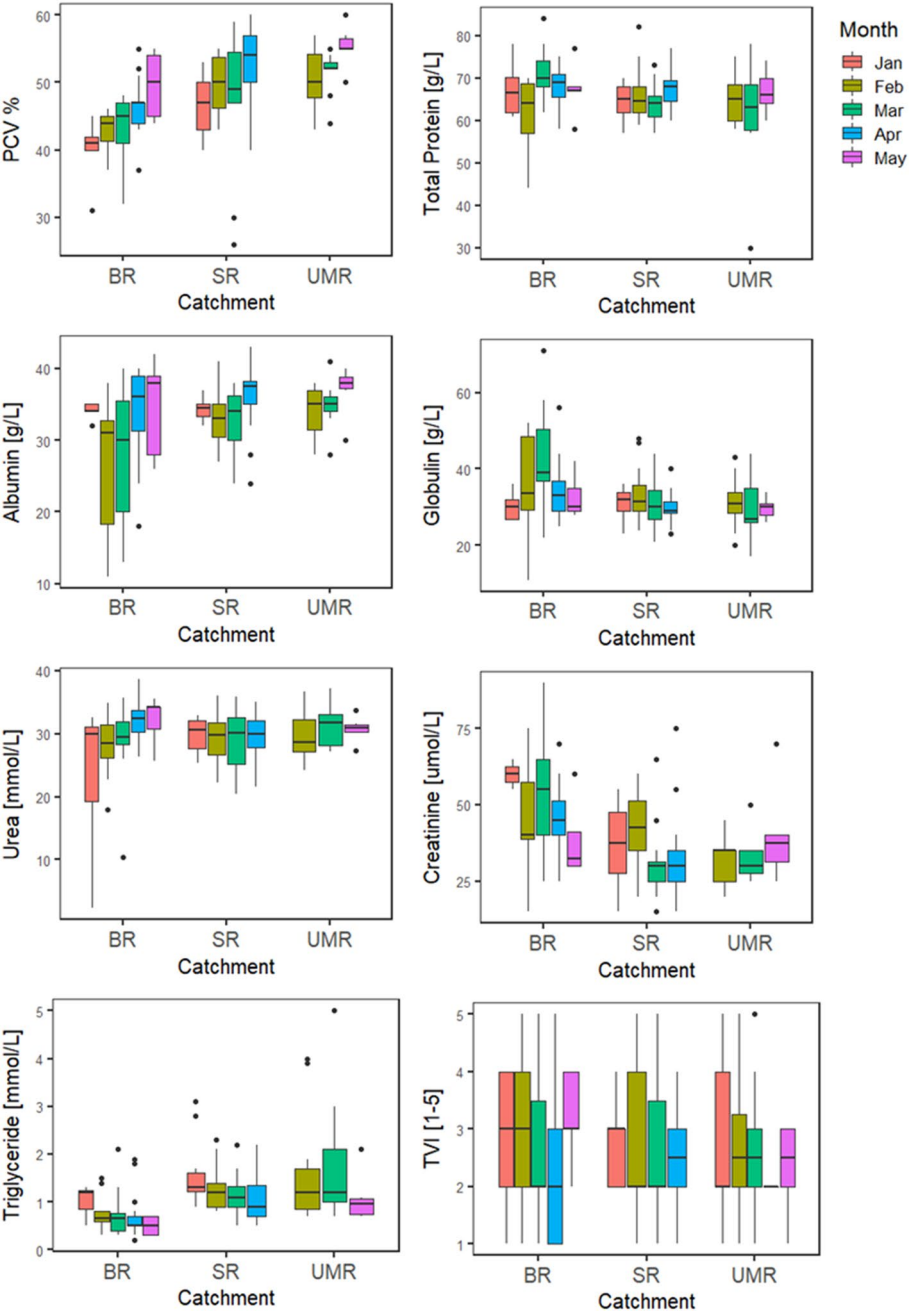
Box plots of seven hematology and serum chemistry analytes of platypuses from all rivers (*BR* Border Rivers n = 83, *SR* Snowy Rivers n = 88, *UM* Upper Murray Rivers n = 38, January to May), collected between January 2016 and May 2018. Boxes represent the 2nd and 3rd quartiles with median represented by the line and whiskers extend to the upper and lower quartiles no further than 1.5 IQR.

**Figure 3:**
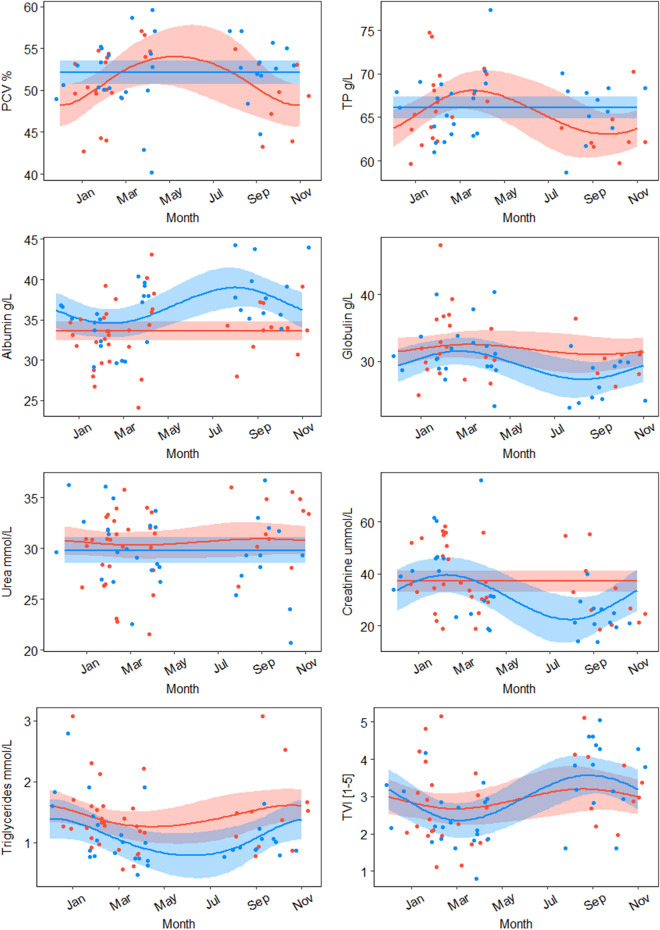
Predicted responses from Generalized Additive Models in seven hematology and serum chemistry analytes and body condition (Tail Volume Index) of female (red) and male (blue) platypuses between December 2016 and November 2017 in the Snowy River Catchment.

Tail volume index (TVI) was not significantly associated to PCV or any of the tested serum chemistry analytes (Appendix S1), although there was some suggestive negative association with levels of triglycerides (*P*  = 0.074) and urea (*P*  = 0.064). Across the three catchments, TVI was not associated with month (Jan-May), sex, age, or elevation (Fig. 2, Appendix S1). On the Snowy River, TVI did not differ between the sexes, but did significantly fluctuate in male platypuses (*P*  = 0.001) and marginally in females (*P*  = 0.086), increasing towards the onset of the breeding season (October) and reaching a minimum by April (Fig. 3, Appendix S2).

All hematology and serum chemistry analytes, apart from urea, were significantly related to catchment (Fig. 2, Appendix S1). Platypuses from the Border Rivers catchment had lower PCV (*P*  = 0.001), and lower concentrations of albumin (*P*  = 0.006), and triglycerides (*P*  = 0.001), compared to those from the Snowy and Upper Murray Rivers catchments (Fig. 2, Appendix S1). Also, TP (*P*  = 0.02), globulin (*P*  = 0.001), and creatinine (*P*  = 0.005) concentrations were significantly higher in platypuses from the Border Rivers catchment, compared to the Snowy and Upper Murray Rivers catchments (Fig. 2, Appendix S1). Urea levels did not vary significantly among rivers, although there was some evidence for lower concentrations in platypuses from the Snowy Rivers catchment, compared to individuals from the other two catchments (*P*  = 0.087), (Appendix S1). Platypuses in the geographically proximal Snowy and Upper Murray River catchments (Fig. 1) had similar levels for all hematology and serum chemistry analytes (Table S1). Further, no hematology nor serum chemistry analytes were related to elevation (Appendix S1).

PCV, and concentrations of TP, albumin, and urea were positively (max *P*  = 0.02) associated with month (Jan to May), while triglycerides concentrations were negatively associated (*P*  = 0.001), and creatinine concentrations were not related to month (*P*  = 0.226) (Fig. 2, Appendix S1). Globulin had a slight negative association with month (*P*  = 0.07). Over a period of a year in the Snowy River catchment, there were no significant fluctuations in concentrations of albumin, urea or triglycerides (Appendix S2). In contrast, PCV (*P*  = 0.03), TP (*P*  = 0.01), and globulin (*P*  = 0.01) concentrations of females in the Snowy Rivers catchment varied significantly over a year (Fig. 3, Appendix S2): all lowest at the end of winter but PCV peaked mid-winter and TP and globulin were highest at the end of summer (Fig. 3). Male globulin also varied significantly (*P*  = 0.03), but had highest globulin levels in autumn and decreasing until their lowest levels in spring (Fig. 3). Male creatinine also fluctuated over the year (*P*  = 0.07) following a similar pattern that of globulin (Fig. 3).

Triglyceride concentrations were lower in males than females (*P*  = 0.001), across all rivers between January to May (Table S1), but there were no other significant differences between the sexes across all rivers. PCV (*P*  = 0.001), TP (*P*  = 0.006) and globulin (*P*  = 0.02) concentrations were higher in males than females in Snowy River platypuses, throughout the year (Fig. 3, Appendix S2). There were no significant differences between the sexes for concentrations of creatinine, albumin, urea, and triglycerides in the Snowy River throughout the year (Appendix S2). Juvenile platypuses had significantly lower PCV (*P*  = 0.002) and lower concentrations of TP (*P*  ≤ 0.001), than adults across all rivers. Globulin was also lower in juveniles but only slightly associated (*P*  = 0.08), (Appendix S1), but there were no significant differences between juveniles and adults for any of the other hematology and serum chemistry analytes.

**Figure 4:**
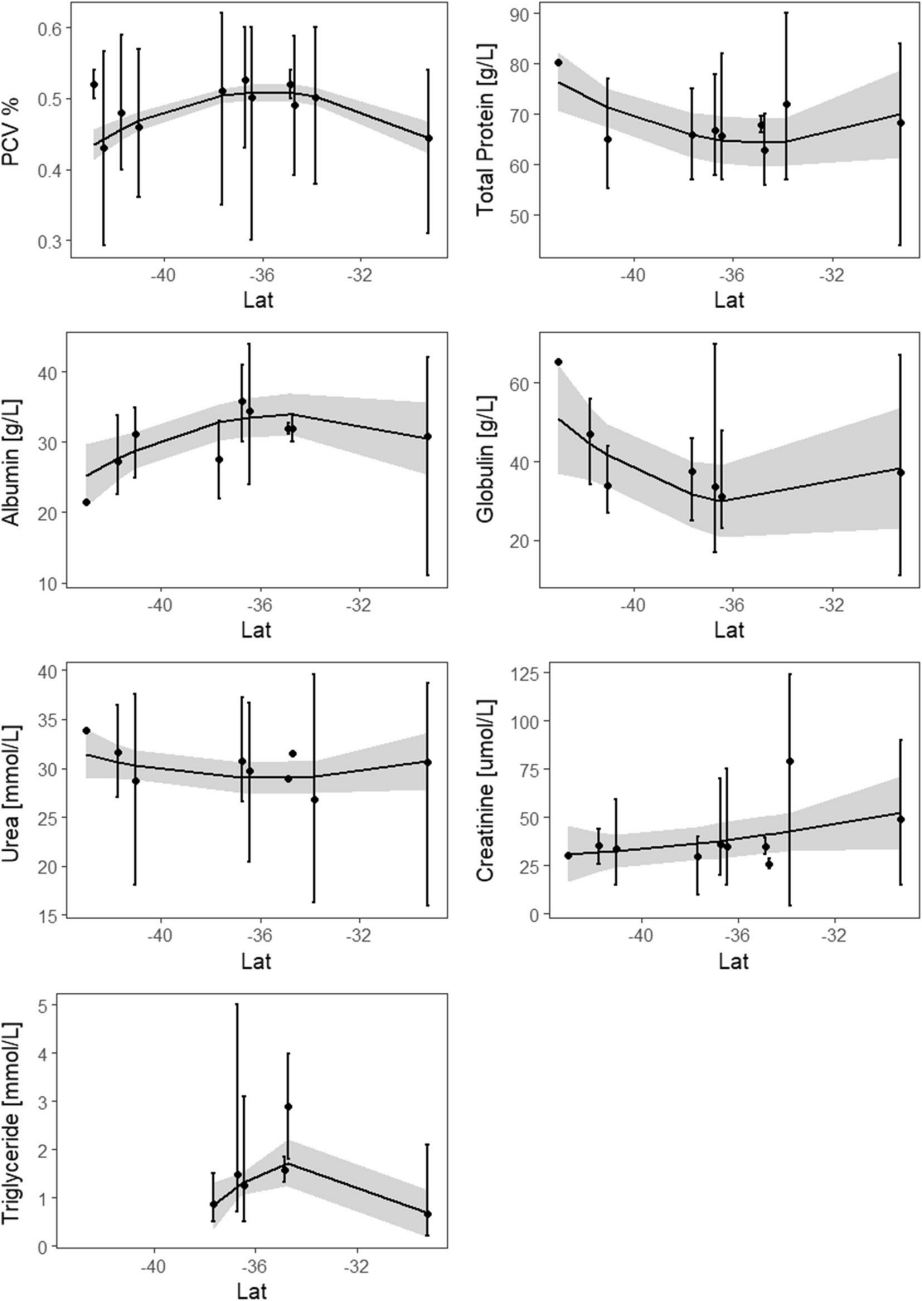
Data from meta-analysis comparing variation (point plot with generalized linear model fitted to data) in levels of seven hematology and serum chemistry variables by latitude.

When all available data from northern New South Wales (Lat -29.24) to Tasmania (Lat -43.05) were analyzed (Figs. 1 & 4, Table 1, Appendix S3), ranges of hematology and serum chemistry analytes in platypus from this study were slightly higher than those of platypus from Tasmania (Table 2). Albumin and PCV decreased with latitude (*P*  = 0.03 and *P*  = 0.001), TP and Triglycerides also showed a slight association (*P*  = 0.06 and *P*  = 0.08), triglycerides however increased with latitude. There were no other significant associations between latitude and the hematology and serum chemistry analytes.

**Table 2:** The seven hematology and serum chemistry variables (sample size), Reference Intervals (RI) (where N ≥ 120) using non-parametric methods^98,99^, mean, SD, and lower and upper 90% confidence intervals, partitioned by age and sex, for platypus captured from New South Wales and Victorian catchments (Snowy, Upper Murray, and Border Rivers) (see Fig. 1).

Group	Variable (n)	RI	Mean	SD	90% CI lower limit	90% CI upper limit
Adults	PCV (191)	33.7–61.8	49	6.6	30–40	58–78
	TP (188)	46.5–78	66.2	6.6	30–55	75–84
	Albumin (184)	14.7–42.4	33.3	6	11–18	40–44
	Urea (184)	19.62–36.73	29.98	4.52	2.3–22.8	36.1–38.7
	Creatinine (161)	15–75	38.8	16.2	15–20	70–90
	Triglycerides (169)	0.3–3.1	1.12	0.69	0.2–0.3	2.3–5
	Globulin (175)	23–57.5	33.8	8.5	11–24	51–71
Juveniles	PCV (44)	27.4–55	44	5.6	26–40	54.8–55
	TP (44)	33–74.9	63.1	7.1	30–57	72–75
	Albumin (42)	18.3–40.9	33.6	5.1	18–24.2	38.9–41
	Urea (42)	21.74–34.94	29.38	3.74	21.7–22.78	33.8–35
	Creatinine* (37)	15.4–62	37.6	11.1	12–20.5	55–66.5
	Triglycerides* (37)	0.32–2.52	1.06	0.57	0.28–0.41	1.95–3.04
Males	PCV (97)	30.9–62.8	49	7.1	30–37.5	59–70
	TP (94)	53.1–76.6	66.4	5.1	47–57.7	74.6–78
	Albumin (91)	15.3–44	33.5	6.5	13–18	40–44
	Urea (92)	17.46–37.07	289.74	4.86	2.3–24.07	36.38–38.7
	Creatinine (78)	15–75	38.8	16.4	15–19.9	70–75
	Triglycerides (85)	0.3–2.66	0.96	0.54	0.2–0.3	1.77–4
	Globulin (89)	20.8–57.5	33.1	8.2	17–23	51–58
Females	PCV (84)	40–63.1	49	6.1	31–42	57–78
	TP (85)	44.2–81.4	66.1	8	30–48.9	76.7–84
	Albumin (94)	13.4–41.3	33	5.5	11–24	40–43
	Urea (94)	18.9–36.19	30.22	4.18	10.4–23.59	35.46–38.7
	Creatinine (83)	15–74.5	38.9	16.1	15–20	65–90
	Triglycerides (84)	0.3–3.8	1.28	0.78	0.3–0.5	2.5–5
	Globulin (78)	22.7–51.5	34.2	7.9	11–26	46.6–71

*Robust method with Cox-Box transformation used due to sample size < 40.

## Discussion

We evaluate several parameters relating to platypus health associated with energy and protein intake by analyzing variation in seven condition-related hematology and serum chemistry analytes. Our study is the largest scale investigation to date of platypus health for mainland platypuses, spanning three different river catchments in New South Wales and Victoria. Measurement of platypus tail volume index, often used to measure condition^85^ was not particularly correlated to the body condition variables in this study. Significant differences occurred across river catchments, between sexes and ages, and among months. Understanding natural variation and impacts of stressors is important for individual and population health assessments^55^ and identifying vulnerabilities in species^33,42,56,102–104^.

All hematology and serum chemistry analytes, except urea, varied significantly between river catchments, with the Border Rivers being significantly different to the other two catchments (Appendix S1, Fig. 2). While our original intent was to create reference intervals for mainland platypus populations, requiring at least 120 samples^57,58^, clear differences in levels in platypuses within the Border Rivers catchment suggested developing a single reference interval across all three studied areas was not appropriate. Consequently, we also calculated confidence intervals for each catchment separately (Table 1), providing geographic comparisons. The relatively lower levels of PCV, albumin and triglycerides, in addition to higher creatinine and globulin levels in platypuses from the Border Rivers catchment, compared to the other two catchments in the species’ southern extent, (Fig. 2, Table 1), may indicate poorer health^44,47,50^, possibly a result of lower food availability, but may also reflect other environmental effects. The significant differences in evaluated hematology and serum chemistry analytes in platypuses in the Border Rivers catchment, the most northerly river catchment surveyed (Fig. 1) may reflect naturally lower levels associated with the climate, indicative of possible habitat-related stressors or some combination of both^105–107^. The sensitivity of platypuses to high temperatures, with limited cooling options^108–111^ is likely a key factor limiting their distribution into the lowland parts of the rivers of the Murray-Darling Basin, despite being widely distributed in connected upper parts (Fig. 1)^78,112–114^. Thermal stress can directly impact health, and lead to changes in hematology and serum chemistry analytes^105,106,115–117^. In addition, indirect effects on health may be caused by changes in temperature shifting food sources and availability^18^. Platypuses rely on aquatic macroinvertebrates for food and are particularly sensitive to changes in temperature regimes^118–121^. Ephemeroptera and Trichoptera have been found to be some of the more sensitive prey to habitat changes^122^ and are also two of the three most common consumed orders by platypus^123^ Understanding the potential risks associated with thermal stress will increase in importance in coming decades, with the northern range of the platypus projected to decrease in size by 31% by 2070 in response to global warming^75^. Thermal stress on the northern populations should be tested with more data from platypuses in Queensland, which is currently poorly sampled^78,124^. Using all available data, latitudinal effects were detected in albumin and PCV, and to some extent with TP and triglycerides (Fig. 4, Table 1). However, variability and reporting deficiencies likely confounded our ability to draw broad conclusions on geographic variation. Collecting more information on platypuses in the species’ northern range (northern New South Wales and Queensland) is needed to evaluate whether observed differences in the Border Rivers were due to geographic variation, habitat stressors, or thermal stress.

Beyond the notable seasonal variation (i.e., month), other potential confounders were present. Methods of restraint varied across studies with some using no anaesthetic and others using different types of anaesthetic (e.g. ether, isoflurane). Anaesthetics can decrease PCV in some species^125–127^, while the stress of handling non-anaesthetised animals can increase PCV^128–131^. In addition, studies differed in blood collection methods and whether PCV or hematocrit was reported^132^. Small sample sets and the inclusion of juveniles in some studies likely further reduced statistical power. This highlights the need for consistent health data collection methods across the species range required to develop population relevant reference intervals. The hematology and serum chemistry analytes for the New South Wales and Victorian populations were similar to those in Tasmania (Table 1, Table 2). Hematology and serum chemistry analytes did not vary across examined elevation gradients, but most varied between the months of Jan and May (Table S2), as well as throughout the year on the Snowy River (Table S3), with some differentiation between the sexes.

There were also seasonal patterns in hematology and serum chemistry, not previously reported for the mainland, such as low levels of triglycerides in winter which may be due to reduced food resources in winter, coinciding with relatively low weight levels^133,134^, and when glucocorticoids are at their highest as animals mobilize energy stores^135,136^. Levels of other hematology and serum chemistry analytes also changed with reproductive status and sex, during the breeding season from August- March^64,66^. Packed Cell Volume in Tasmanian platypuses^81^ were significantly correlated to water and ambient temperature and time of year, increasing from autumn through winter, then decreasing from spring to summer. However, this study found PCV was consistent throughout the year for males (Fig. 3), but females varied with highest levels at the start of winter then decreasing through autumn to the lowest levels throughout summer (Fig. 3). This reflected the considerable metabolic demand on breeding females from construction of nesting burrows in September to October, then egg laying and incubation, followed by four months of lactation before young emerge between January to late February^64,137–139^. The metabolic demand of supporting up to three young is considerable^60^, requiring dramatically increased daily food consumption, during lactation, of 90–100% of their bodyweight^140–142^. Increased PCV in females during breeding times is also evident in other species^143,144^. A similar pattern was observed for TP and globulin (Fig. 3), indicating that variances in blood protein concentrations may be associated with egg production as seen in other egg-laying species^45,145,146^. PCV and serum chemistry concentrations of males were relatively more stable than those in females in the winter months. Patterns of variance were similar between the sexes, however males slightly lagged behind females; lowest values for males were observed early in the breeding season around September for males, while values for females were lowest around December to February (Fig. 3). During the breeding season males may engage in territorial and aggressive behaviour, potentially utilizing their venomous spurs^89,147^, coinciding with increased testosterone production^89,148,149^. Testosterone production negatively affects fat stores and metabolism in other species^150^. Male platypuses in captivity were more active than females in the pre-mating season (May to August) and the post-mating season (October to January)^151,152^, with most of their time spent defending territory from other males or seeking out females rather than foraging. This may explain the lower concentrations observed in males during the breeding season. Detection of monthly patterns in hematology and serum chemistry were weakened because of the absence of sampling in May–July, although these monthly patterns were still evident in the data (Fig. 3).

The effect of age on hematology and serum chemistry can be seen in many marsupials^17,153–159^ and has been linked to changes in body mass, ontogenesis of the immune system^160^, energy requirements for growth, and changes in behaviour. For example, juvenile platypuses often disperse further than adults, particularly males^133^. Packed Cell Volume and TP concentrations generally increase with age, as seen with juveniles in this study having significantly lower PCV, TP and globulin compared to adults (Appendix S1).

In conclusion, we investigated PCV and six serum chemistry analytes and found a number of seasonal changes and associations with river catchment, sex and age. The causes of spatial differences remain largely unknown, a reflection of natural variability and the range of environmental stressors affecting platypus populations^72,73,75–78,161^. Further investigations incorporating direct measures of health provided by hematology and serum chemistry analytes in conjunction with indirect measures of body condition are warranted with expansion of the number of analytes measured (Table 2). Measurement of hematology and serum chemistry analytes may provide more comprehensive information on an animal’s health than indirect measures alone^55,107^. While we acknowledge that this study used a subset of hematology and serum chemistry analytes, given the association of these analytes to energy, protein intake, body mass and organ function, we suspect TVI may not provide an accurate measure of health but nonetheless reflective of fat storages which fluctuate seasonally^85,162^. Although a slight negative association was seen in urea and triglycerides, warranting further investigation as an indicator of body condition^28,162,163^. There is also a need to better understand spatial relationships between hematology and serum chemistry analytes and the range of threatening processes, varying across the distribution of the platypus^72,124,164^. There are still considerable knowledge gaps in understanding of platypus ecology and biology across their range, with this compilation of data providing the first large-scale assessment of condition for mainland platypuses, including reference intervals for selected hematology and serum chemistry analytes for the most northern population to date. Measurement of these variables in platypus can continue to improve current understanding of the ecology of this scientifically valuable and irreplaceable part of Australian and global biodiversity, contributing to its future survival.

## Supplementary Information

Supplementary Information.

## Data Availability

Data is available through Dryad (10.5061/dryad.brv15dv9d).
